# Neonatal assessment in the delivery room – Trial to Evaluate a Specified Type of Apgar (TEST-Apgar)

**DOI:** 10.1186/s12887-015-0334-7

**Published:** 2015-03-08

**Authors:** Mario Rüdiger, Nicole Braun, Jacob Aranda, Marta Aguar, Renate Bergert, Alica Bystricka, Gabriel Dimitriou, Khaled El-Atawi, Sascha Ifflaender, Philipp Jung, Katarina Matasova, Violeta Ojinaga, Zita Petruskeviciene, Claudia Roll, Jens Schwindt, Burkhard Simma, Nanette Staal, Gloria Valencia, Maria Gabriela Vasconcellos, Maie Veinla, Máximo Vento, Benedikt Weber, Anke Wendt, Sule Yigit, Heinz Zotter, Helmut Küster, The TEST-Apgar study-group

**Affiliations:** Department of Neonatology and Pediatric Intensive Care, Medizinische Fakultät Carl Gustav Carus, TU Dresden, 01307 Dresden, Germany; Department of Pediatrics, State University of New York Downstate Medical Center, Brooklyn, NY 11203 USA; Division of Neonatology, University & Polytechnic Hospital La Fe, 46009 Valencia, Spain; Faculty Hospital, Department of Neonatology, 94001 Nové Zámky, Slovakia; Neonatal Intensive Care Unit, Department of Pediatrics, University of Patras Medical School, Rio Patras, 26500 Greece; Neonatal Intensive Care Unit, Department of Pediatrics, Latifa Hospital, Dubai Health Authority, Government of Dubai, United Arab Emirates; Clinic for Pediatrics, University Hospital Schleswig-Holstein, Campus Lübeck, 23562 Lübeck, Germany; Clinic of Neonatology, Jessenius Faculty of Medicine Martin, Comenius University Bratislava, University Hospital in Martin, 03601 Martin, Slovakia; División de Neonatología, Instituto Nacional de Perinatología, 11000 México, D.F México; Department of Neonatology, Kaunas University of Medicine, Kaunas, 44307 Lithuania; Department of Neonatology and Pediatric Intensive Care, Vest Children’s Hospital Datteln, University Witten/Herdecke, 45711 Datteln, Germany; Department of Pediatrics and Adolescent Medicine, Division of Neonatology, Pediatric Intensive Care and Neuropediatrics, Medical University Vienna, 1090 Vienna, Austria; Department of Pediatrics, University Teaching Hospital, 6807 Feldkirch, Austria; Department of Neonatology, Hospital S. João, 4200 – 319 Porto, Portugal; Department of Neonatology, Children’s Clinic of Tartu University Hospital, 51014 Tartu, Estonia; Clinic for Neonatology, University Hospital Charité Berlin, Campus Virchow, 13353 Berlin, Germany; Clinic for Neonatology, University Hospital Charité Berlin, Campus Mitte, 10117 Berlin, Germany; Department of Pediatrics, Hacettepe University Faculty of Medicine, 06100 Ankara, Turkey; Department of Pediatrics, Division of Neonatology, Medical University of Graz, 8036 Graz, Austria; Children’s Hospital, University of Göttingen, 37099 Göttingen, Germany

**Keywords:** Apgar score, Expanded-Apgar, Specified-Apgar, Postnatal condition, Neonatal assessment, Delivery room management, Preterm resuscitation

## Abstract

**Background:**

Since an objective description is essential to determine infant’s postnatal condition and efficacy of interventions, two scores were suggested in the past but weren’t tested yet: The Specified-Apgar uses the 5 items of the conventional Apgar score; however describes the condition regardless of gestational age (GA) or resuscitative interventions. The Expanded-Apgar measures interventions needed to achieve this condition. We hypothesized that the combination of both (Combined-Apgar) describes postnatal condition of preterm infants better than either of the scores alone.

**Methods:**

Scores were assessed in preterm infants below 32 completed weeks of gestation. Data were prospectively collected in 20 NICU in 12 countries. Prediction of poor outcome (death, severe/moderate BPD, IVH, CPL and ROP) was used as a surrogate parameter to compare the scores. To compare predictive value the AUC for the ROC was calculated.

**Results:**

Of 2150 eligible newborns, data on 1855 infants with a mean GA of 28^6/7^ ± 2^3/7^ weeks were analyzed. At 1 minute, the *Combined-Apgar* was significantly better in predicting poor outcome than the *Specified-* or *Expanded-Apgar* alone. Of infants with a very low score at 5 or 10 minutes 81% or 100% had a poor outcome, respectively. In these infants the relative risk (RR) for perinatal mortality was 24.93 (13.16-47.20) and 31.34 (15.91-61.71), respectively.

**Conclusion:**

The Combined-Apgar allows a more appropriate description of infant’s condition under conditions of modern neonatal care. It should be used as a tool for better comparison of group of infants and postnatal interventions.

**Trial registration:**

clinicaltrials.gov Protocol Registration System (NCT00623038). Registered 14 February 2008.

**Electronic supplementary material:**

The online version of this article (doi:10.1186/s12887-015-0334-7) contains supplementary material, which is available to authorized users.

## Background

An objective assessment of infant’s postnatal condition in the delivery room is essential for clinical care and scientific purposes. To describe the postnatal condition of groups of infants or to compare effects of interventions in a research setting a numerical score, which represents the sum of several objective findings is required.

To describe the condition of a newborn, Virginia Apgar developed a system that scores the postnatal condition by converting clinical observations into quantifiable scientific data [1]. However, there is no general agreement on how to score infants with a low gestational age or those receiving interventions [2-4]. To overcome that problem, we suggested to specify the items of the conventional Apgar and to score infant’s condition regardless of gestational age and interventions (*Specified-Apgar*) [5]. According to the rules of the *Specified-Apgar*, the full score with a maximum total of 10 points can either be allocated to the healthy term or preterm infant without any problems in postnatal adaptation, but also to an infant receiving resuscitative or supportive interventions with an adequate response to those interventions (good chest expansion during ventilation, pink skin colour due to supplemental oxygen, etc.). To better differentiate between both conditions, the American Academy of Pediatrics (AAP) and American College of Obstetricians and Gynecologists (ACOG) suggested to also score and document the interventions that are required to achieve the condition (*Expanded-Apgar*) [6]. Consequently, an infant without any interventions would have a higher *Expanded-Apgar* than the one who requires interventions. It can be assumed that an infant’s condition is better described using both scores (*Combined-Apgar*) simultaneously than one score alone.

Up until now, both scores are not used in clinical practise mainly because of not being validated yet.

The multicenter, international TEST-Apgar study (“**T**rial to **E**valuate a **S**pecified **T**ype of **Apgar**”) aimed to answer the question, whether the *Combined-Apgar* describes infant’s postnatal condition better than either the *Specified-Apgar* or *Expanded-Apgar* alone. Since no “gold standard” was available, we decided to use prediction of mortality and morbidity as a proxy for testing. That outcome criterion was only used as a surrogate parameter for the purpose of testing the scores. It was not the aim to develop a new tool which predicts mortality or morbidity, but to test that the *Combined-Apgar* provides a good numerical description of infant’s postnatal condition.

Therefore, the primary hypothesis of the TEST-Apgar study was as follows: the *Combined-Apgar* is a better predictor of poor outcome in preterm infants (defined as either death or any major morbidity during the first hospital stay) than either the *Specified-Apgar* or *Expanded-Apgar* alone.

## Methods

In an observational study data were collected prospectively in 20 academic neonatal intensive care units (NICU) in 12 countries from March 2008 to June 2009.

### Inclusion and exclusion criteria

Infants were eligible if they were born at any of the study sites prior to 32 completed weeks of gestation. The exclusion criteria were: 1.) lack of informed consent, 2.) outborn, 3.) any major congenital malformation, or 4.) death in the delivery room.

### Data

After birth, the following data were collected by the attending neonatologist: gestational age, birth weight, mode of delivery and a description of the infant at 1, 5 and 10 minutes of life according to the definition of the *Combined-Apgar*, which consists of the *Expanded-Apgar* and *Specified-Apgar* as shown in Table 1:

**Table 1:** ***Combined-Apgar*** **consisting of the** ***Expanded-Apgar*** **and** ***Specified-Apgar***

		**Minute**		
		**1**	**5**	**10**
**C**	CPAP^#^			
**O**	Oxygen Supplementation			
**M-B**	Mask and Bag Ventilation^##^			
**I**	Intubation and Ventilation			
**N**	Neonatal Chest Compression			
**E**	Exogenous Surfactant Administration			
**D**	Drugs			
0 = intervention was performed				
1 = no intervention				
# Score 0, if ‘Mask and Bag Ventilation’ or ‘Intubation and Ventilation is scored 0.				
## Score 0, if ‘Intubation and Ventilation’ is scored 0.				
**Sum of the** ***Expanded-Apgar*** **:**				
Skin Color*	2 = completely pink			
**A**	1 = centrally pink with acrocyanosis			
	0 = centrally blue or pale			
Heart Frequency*	2= >100/min			
**P**	1 = 100-1/min			
	0 = no heart beat			
Reflex	2 = appropriate for gestational age			
**G**	1 = reduced for gestational age			
	0 = no reflex responses			
Muscle Tone	2 = appropriate for gestational age			
**A**	1 = reduced for gestational age			
	0 = completely flaccid			
Chest Movement*	2 = regular chest movement			
**R**	1 = small or irregular chest movement			
	0 = no chest movement			
*Independent of the requirements needed to achieve this condition				
**Sum of the** ***Specified-Apgar*** **:**				
**Total (** ***Expanded- + Specified-Apgar*** **):**

1. *Specified-Apgar* [5]: Muscle tone and reflex response were evaluated in relation to GA as being appropriate (2 points), reduced (1 point) or absent (0 points). Chest movement was evaluated regardless of the respiratory support given and was scored 2 if chest movements were appropriate, with 1 point if chest movements were reduced, irregular or signs of respiratory distress were present, and scored 0 if no chest movement was present. Skin color and heart frequency were evaluated as detailed in Table 1, regardless of the intervention needed to achieve this condition.
2. *Expanded-Apgar* [6]: Presence or absence of the following interventions was evaluated: continuous positive airway pressure (CPAP), oxygen supplementation, bag and mask ventilation, intubation and ventilation, chest compression, administration of surfactant, epinephrine (drugs). If an intervention was performed it was scored 0, if absent it was scored 1. The best possible score was 7 (no intervention) and the worst 0 (all interventions performed). Intubated and ventilated infants were scored 0 for CPAP as well as for bag and mask ventilation; infants on bag and mask ventilation were scored 0 for CPAP as well.
3. *Combined-Apgar:* In clinical routine, the *Combined-Apgar* will consist of two numbers, such as 7–10 for the *Expanded-* and *Specified-Apgar,* respectively (Table 1). For the purpose of the present study, the Combined-Apgar was calculated as the sum of the *Specified-* and the *Expanded-Apgar*.

At discharge the following outcome measures were recorded: mode of discharge (death, transfer to another hospital, or home), length of stay, corrected GA, weight, and the presence of either of the four major morbidities: moderate/severe bronchopulmonary dysplasia (BPD) [7], intraventricular haemorrhage grade 1–4 (IVH), cystic periventricular leukomalacia (CPL), and retinopathy of prematurity (ROP).

### Data management and statistical analyses

Participating doctors were instructed regarding definitions and study forms prior to the start of the study at the individual center. Data collected at participating sites were transmitted to the principal investigator and analyzed for the primary outcome criterion consisting of death or either one of the major morbidities (BPD, IVH, CPL, ROP). To test the hypothesis, the predictive values of the scores were compared by calculating the areas under the curve (AUC) and their co-variances of the receiver-operating-characteristics (ROC), based on an algorithm given by DeLong and co-workers [8]. The comparisons of AUCs base on WALD tests. The significance of differences between estimated risks was tested by Chi-square tests; p-values are unadjusted for multiple testing. For sample size calculation a difference in AUC means of 0.01 and a SD in matched pairs of 0.1 were assumed. To detect a significant difference at a level of p = 0.01 with a power of 0.9 data of 1490 infants were required. Assuming a dropout rate of 30%, it was planned to recruit 2000 patients. For secondary data analysis, the AUC was calculated for each individual component of poor outcome. Data on mortality were analyzed for “death at any point” and “perinatal mortality”. Furthermore, for each outcome criteria the relative risk (RR) and 95% confidence interval (95%-CI) were calculated. To do so, scores were categorized as follows: *Specified-Apgar:* poor (0–3 points), fair (4–6 points) or good (7–10 points) [9], *Expanded-Apgar:* low (0–2 points), moderate (3–4 points), high (5–6 points) or no intervention (7 points); *Combined-Apgar:* very low (0–5 points), low (6–9 points), moderate (10–13 points) or high (14–17 points). The respectively highest category of each score was used as reference value. All analyses were performed by using SAS (SAS Institute Inc., Cary, NC, version 9.2).

### Ethics committee approval

Approval was given by the Ethics Committee of the Medical Faculty Carl Gustav Carus, Dresden, Germany (EK 104052008) and in all participating centers. Written informed consent was obtained from the infant’s parents before taking part in the study. The study was registered on ClinicalTrials.gov Protocol Registration System (NCT 00623038).

## Results

### Patient characteristics

In total, 2150 infants were eligible for the study. Two hundred ninety five had to be excluded from analysis for the following predetermined reasons: Missing parental consent (n = 28), outborn (n = 24), congenital anomalies (n = 16), palliative care (n = 16), transferral within first week of life (n = 3), no paediatrician present (n = 2), incomplete data (n = 143), other reasons (n = 63). Perinatal data of excluded patients (mean GA of 28^3/7^ ± 2^3/7^ weeks, birth weight 1119 ± 388 g) were statistically not different from those of analyzed patients.

Data of 1855 patients were analyzed, representing in median 89 (Range 21–228) patients per center with a mean GA of 28^6/7^ ± 2^3/7^ weeks and birth weight of 1172 ± 409 g.

The overall mortality rate was 10.6%. For the individual centers, the median mortality rate was 8.8% [Interquartile Range (IQR) 4.8-12.3%]. Infants were discharged home at a median postconceptional age of 36^2/7^ weeks (length of stay in survivors 53 [IQR 35–75] days).

### Gestational age – effect on clinical condition and need for medical interventions

**Figure 1:**
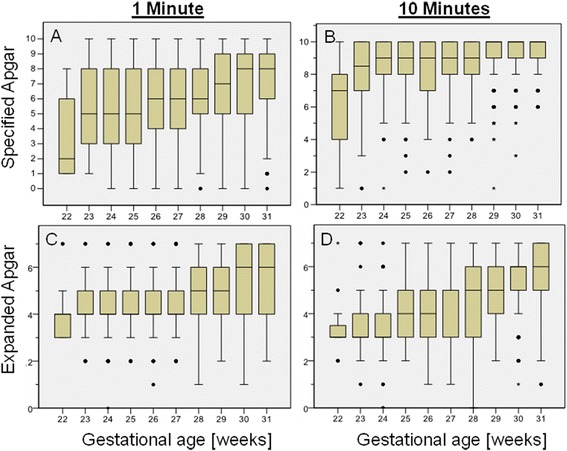
**Distribution-pattern of the Specified-Apgar (A, B) and Expanded-Apgar (C, D) at 1 and 10 minutes.** In the boxplot, the central box represents the values from the lower to upper quartile (25 to 75 percentile). The middle line represents the median. The vertical line extends from the minimum to the maximum value. Outlier values are displayed as black dots, extreme values are displayed as an asterix.

The postnatal condition during the first minute of life showed a linear correlation with gestational age; with the *Specified-Apgar* scoring higher at higher gestational age (Figure 1A). In contrast, there was no linear correlation between gestational age and medical interventions *(Expanded-Apgar)* at one minute; medical support was almost similar for infants below 28 weeks of gestation (Figure 1C).

At 10 minutes, clinical condition of all infants was similar regardless of gestational age (Figure 1B). However, medical support to achieve this condition increased with decreasing gestational age (Figure 1D).

### Primary outcome measure – prediction of poor outcome

To test the primary hypothesis, the AUC of ROC-curves for prediction of poor outcome were calculated and compared (Table 2). At 1 minute, the *Combined-Apgar* predicts poor outcome significantly better than the *Specified*- or *Expanded-Apgar* alone. At 5 and 10 minutes, the *Combined-Apgar* predicts poor outcome better than the *Specified-Apgar*. However, there were no significant differences in predicting poor outcome between *Combined*- and *Expanded-Apgar* at 5 and 10 minutes.

**Table 2:** **Area under the curve (AUC) of the ROC-Curve for the** ***Specified-Apgar*** **,** ***Expanded-Apgar*** **and** ***Combined-Apgar***

		**Specified-Apgar**	**p-value**	**Expanded-Apgar**	**p-value**	**Combined-Apgar**
		**AUC (95%-CI)**		**AUC (95%-CI)**		**AUC (95%-CI)**
Poor Outcome	1 Minute	0.63 (0.59 to 0.68)	<.001	0.64 (0.60 to 0.68)	0.007	0.66 (0.62 to 0.70)
	5 Minutes	0.63 (0.59 to 0.67)	<.001	0.70 (0.65 to 0.74)	1.00	0.69 (0.65 to 0.74)
	10 Minutes	0.61 (0.56 to 0.65)	<.001	0.70 (0.65 to 0.75)	1.00	0.70 (0.65 to 0.75)
Morbidity in survivors	1 Minute	0.58 (0.54 to 0.61)	<.001	0.58 (0.55 to 0.62)	0.61	0.59 (0.55 to 0.63)
	5 Minutes	0.57 (0.53 to 0.60)	<.001	0.63 (0.59 to 0.67)	0.002	0.62 (0.58 to 0.66)
	10 Minutes	0.55 (0.51 to 0.59)	<.001	0.64 (0.60 to 0.68)	0.008	0.63 (0.59 to 0.67)
Death	1 Minute	0.68 (0.64 to 0.71)	<.001	0.67 (0.64 to 0.71)	<.001	0.70 (0.67 to 0.74)
	5 Minutes	0.68 (0.65 to 0.72)	<.001	0.70 (0.66 to 0.74)	<.001	0.73 (0.69 to 0.77)
	10 Minutes	0.67 (0.63 to 0.70)	<.001	0.68 (0.64 to 0.72)	<.001	0.71 (0.67 to 0.75)
Perinatal mortality	1 Minute	0.73 (0.69 to 0.76)	<.001	0.71 (0.68 to 0.75)	<.001	0.76 (0.72 to 0.80)
	5 Minutes	0.72 (0.69 to 0.76)	<.001	0.74 (0.70 to 0.77)	<.001	0.78 (0.74 to 0.82)
	10 Minutes	0.71 (0.67 to 0.74)	<.001	0.73 (0.70 to 0.77)	<.001	0.77 (0.73 to 0.81)

Shown are the values for the Area under the curve (AUC) and 95%-CI of the ROC-curve for the prediction of poor outcome, morbidity in survivors, death and perinatal mortality by the *Specified-Apgar*, *Expanded-Apgar* and *Combined-Apgar*.

p-values are calculated vs. *Combined-Apgar.*

Analysis of the single components of outcome revealed that overall morbidity in survivors was better predicted by the number of interventions (*Expanded-Apgar*) at 5 and 10 minutes than by the actual condition *(Specified-Apgar)* of the infant at that time (see Additional file 1: Table A and Additional file 2: Table E). In contrast, death (especially perinatal mortality) was better predicted by the postnatal condition (*Specified-Apgar*) (see Additional file 2: Table F). The *Combined-Apgar* was significantly better to predict perinatal mortality than the *Specified-* or *Expanded-Apgar* alone (see Additional file 3: Table J; Additional file 2: Table F and Additional file 1: Table B).

### Relative risk for morbidity or mortality for each score

Detailed data on the relative risk for morbidity and mortality can be found as additional files (for the *Expanded-Apgar* see Additional file 1: Table A-D; for the *Specified-Apgar* see Additional file 2: Tables E-H; and for the *Combined-Apgar* see Additional file 3: Tables I-L).

Infants with a persistently poor *Specified-Apgar* (<4) up to minute 5 or minute 10 had a poor outcome in 75% or 93% (RR 1.67 [95%-CI 1.41 to 1.98] or 2.08 [1.80 to 2.40]), respectively. If the five outcome parameters were analyzed separately, the *Specified-Apgar* cannot be used to predict the morbidity risk. Best prediction was found for mortality: the risk of death was significantly increased with a poor and a fair *Specified-Apgar* (see Additional file 2: Table F).

**Figure 2:**
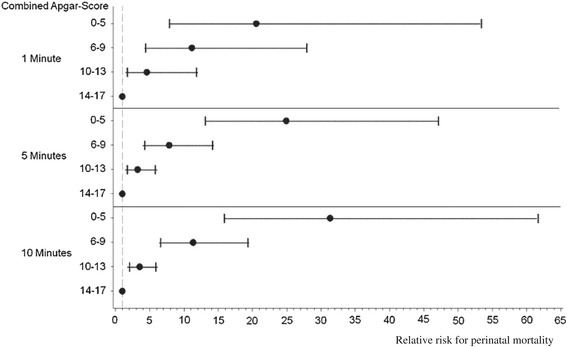
***Combined-Apgar*** **: Relative risk for perinatal mortality.**

In 30% or 24% the *Expanded-Apgar* remained below 5 for up to 5 or 10 minutes, respectively. Less than 1% had an *Expanded-Apgar* below 3 for up to 5 or 10 minutes. The risk of poor outcome was below 30% in infants without any intervention; however, it increased to above 50% with a moderate (score 3–4) and above 60% with a low (score 0–2) *Expanded-Apgar* (see Additional file 1: Table A). For the *Combined-Apgar*, the relative risk of poor outcome increased with a decreasing score (see Additional file 3: Table I). As shown in Figure 2, a very low *Combined-Apgar* was associated with an about 30-fold increased risk for perinatal mortality.

## Discussion

A numerical score that represents the sum of objective findings is a prerequisite to describe the postnatal condition or to compare effects of interventions in the delivery room for groups of infants. Since the infant’s condition is affected by medical interventions, it seems mandatory to describe not only the clinical status but also the interventions needed to achieve this condition. During the last centuries, various attempts have been made to describe the infant’s condition after birth [10-12]. Virginia Apgar developed a scoring system that now bears her name and is widely used [1]. Since improvements in neonatal care demanded a recalibration of the Apgar score [13], the *Specified-Apgar* was introduced. Using the same items as the original score, it allows detailed descriptions for the infant’s condition regardless of GA or interventions [5]. To account for medical interventions required to achieve this condition the AAP and ACOG suggested an expanded reporting form for concurrent resuscitative interventions (*Expanded-Apgar*) [6]. Both scores together give an objective measure of the postnatal condition; however this *Combined-Apgar* has not been used in clinical studies yet. The multicenter international TEST-Apgar study tested both scores for the first time in a clinical setting. The data show that the combination of assessing infant’s condition and administered interventions (*Combined-Apgar*) is better (to predict death) than the description of condition or interventions alone.

The description of the postnatal condition is incomplete without considering administered interventions. The *Expanded-Apgar* adds seven items as a measure for medical interventions needed to achieve the infant’s condition [6]. This extension seems long needed, because care of the newborn has changed significantly and an infant’s condition often depends on medical interventions used to support postnatal transition. In the present study, the majority of infants showed a good clinical condition at 10 minutes of life, however, the number of interventions needed to achieve that condition increased with decreasing gestational age.

The seven items of the *Expanded-Apgar* represent medical interventions that are clearly defined. To our knowledge, this is the first prospective clinical study to show a direct relationship between the number of interventions administered postnatally and subsequent survival. The more interventions were given the more likely the infant died within the first postnatal week. All infants with a *Combined-Apgar* below 6 for at least 10 minutes had a bad outcome. However, at this moment it remains unclear whether all interventions were clinically indicated or rather done as a result of current practice in individual institutions. More detailed analyses will have to analyze if there are differences in the predictive power of individual items of the *Combined-Apgar.*

### Clinical implications

In the past, the 1-minute Apgar score was used to guide further treatment and the 5-minute score was a useful index of the effect(iveness) of resuscitation efforts [14]. However, it remained unclear how infants were scored during resuscitation. It was suggested to interrupt resuscitation for evaluation purposes [3,15] but this approach seems not practical. The *Specified-Apgar* describes the condition without interrupting intervention and the *Expanded-Apgar* adds the required information concerning the interventions. Thus, the *Combined-Apgar* evaluates both, infant’s condition and resuscitative efforts and can be used to guide postnatal interventions.

A *Combined-Apgar* of 7–10 represents an infant without any clinical interventions (*Expanded-Apgar* of 7) and good clinical signs (*Specified-Apgar* of 10). In contrast a *Combined-Apgar* of 0–10 represents an infant with full resuscitative interventions (*Expanded-Apgar* 0) and a good clinical response (*Specified-*Apgar of 10). Finally, a *Combined-Apgar* of 0–0 represents an infant with full resuscitative interventions but no clinical response.

### Limitations of the study

It has been previously shown that not all items of the conventional Apgar score are of equal importance [9,16]. However, Virginia Apgar did not differentially weigh or remove individual items since it was her intention to have a score that can be “determined easily and without interfering with the care of the infant” [1]. The *Combined-Apgar* never intended to replace but rather to specify the score that has been used worldwide for almost 60 years. Therefore, items were neither changed nor omitted. Furthermore, definition of skin color was not changed despite its poor correlation with oxygen saturation [17].

### Questions that were not answered by the present study

Since there is no other objective measure to describe postnatal condition, poor outcome was used as a surrogate parameter to test the value of the scores in preterm infants. It has already been noted by Virginia Apgar that her score cannot be used to predict survival in an individual infant, but only for a group of infants [18,19]. For a better prediction of outcome in individual infants other more reliable scores should be used [19-21]. But it remains unclear, whether this higher predictive value in individuals is of relevance in clinical routine.

Whereas virtually every newborn is evaluated by the Apgar score today, recent studies suggest problems concerning its reproducibility in individual infants [2-4]. A study, comparing conventional Apgar scores assigned by observers of resuscitation videos to those given by the staff attending the delivery, revealed a poor inter-observer reliability [4]. Similarly, a poor reliability was found when Apgar scores were assessed for written case descriptions [3]. In clinics where case descriptions were scored low, preterm infants received lower Apgar scores as well [2]. It was speculated, that this variability “could also be due to variations in the application of the scoring system” [22]. To overcome its poor reproducibility, the *Specified-Apgar* was introduced, which gives more detailed descriptions of the infant’s condition regardless of GA or interventions needed to achieve this condition [5]. Strict definitions – as given by the *Specified-Apgar* – are needed to minimise variability in the description of infant’s condition. However, subsequent studies have to test, whether reproducibility is actually improved by using the *Specified-Apgar*.

Another important practical aspect of the *Combined-Apgar* is its applicability for every newborn. The present study has only tested the predictive power in very preterm infants, but the Apgar score is also of importance in resuscitated infants (e.g. it is used as an inclusion criterion for hypothermia) [23]. Considering the large variation in the conventional Apgar score for an individual infant depending on the care givers opinion [2-4], it could be assumed that the *Combined-Apgar* will be a better discriminator. However, the predictive power of the *Combined-Apgar* for subsequent neurological impairment has to be tested in asphyxiated infants.

## Conclusions

In summary, the present study tested a numerical score (*Combined-Apgar*) that sums up objective findings upon the condition of the infant in the delivery room and the interventions needed to achieve this condition in a large population of preterm infants. The *Combined-Apgar* is a good tool to describe the postnatal condition in the delivery room as shown by its ability to predict perinatal mortality for groups of infants. It should be used in subsequent studies that require a detailed description of infant’s postnatal situation.

### Availability of supporting data

Additional files (Additional files 1, 2 and 3: Tables A-L) are available as supporting data for the *Expanded-*, *Specified*- and *Combined-Apgar* and the relative risk of poor outcome and morbidity in survivors, death and perinatal mortality, BPD and ROP as well as IVH and CPL.

## Additional files

Additional file 1:
***Expanded-Apgar*** **and the relative risk of… (Table A): … poor outcome and morbidity in survivors (page 23), (Table B): … death and perinatal mortality (page 24), (Table C): … BPD and ROP (page 25), (Table D): … IVH and CPL (page 26).**
Additional file 2:
***Specified-Apgar*** **and the relative risk of… (Table E): … poor outcome and morbidity in survivors (page 27), (Table F): … death and perinatal mortality (page 28), (Table G): … BPD and ROP (page 29), (Table H): … IVH and CPL (page 30).**
Additional file 3:
***Combined-Apgar*** **and the relative risk of… (Table I): … poor outcome and morbidity in survivors (page 31), (Table J): … death and perinatal mortality (page 32), (Table K): … BPD and ROP (page 33), (Table L): … IVH and CPL (page 34).**

## Abbreviations

AUC: Area under the curve
BPD: Bronchopulmonary dysplasia
CPL: Cystic periventricular leukomalacia
DR: Delivery room
GA: Gestational age
IVH: Intraventricular haemorrhage
NICU: Neonatal intensive care unit
ROC: Receiver-operating-characteristics
ROP: Retinopathy of prematurity
