# Supplementary material for: Neonatal assessment in the delivery room – Trial to Evaluate a Specified Type of Apgar (TEST-Apgar)
Source: BMC Pediatr. 2015 Mar 8;15:18. doi: 10.1186/s12887-015-0334-7 (PMC4374498; doi:10.1186/s12887-015-0334-7)
Supplement: Additional file 1: — Expanded-Apgar and the relative risk of… (Table A): … poor outcome and morbidity in survivors (page 23), (Table B): … death and perinatal mortality (page 24), (Table C): … BPD and ROP (page 25), (Table D): … IVH and CPL (page 26). [file 12887_2015_334_MOESM1_ESM.pdf]

Additional File 1, Table A. *Expanded-Apgar* and the relative risk for poor outcome and morbidity in survivors

			**Poor Outcome**			**Morbidity in Survivors**		
Expanded-Apgar		**N (%)**	**n (%)**	**RR (95%-CI)**	**P-value**	**n (%)**	**RR (95%-CI)**	**P-value**
**1 minute**	**0-2 points**	54 (3)	36 (67)	2.35 (1.82 to 3.04)	<.001	23 (43)	1.70 (1.19 to 2.45)	0.007
	**3-4**	732 (40)	411 (56)	1.98 (1.65 to 2.38)	<.001	299 (41)	1.63 (1.33 to 2.01)	<.001
	**5-6**	709 (39)	286 (40)	1.42 (1.17 to 1.73)	<.001	237 (33)	1.34 (1.08 to 1.66)	0.006
	**7**	328 (18)	93 (28)	1.00		82 (25)	1.00	
**5 minutes**	**0-2**	74 (4)	59 (80)	3.70 (2.83 to 4.82)	<.001	36 (49)	2.73 (1.91 to 3.90)	<.001
	**3-4**	697 (38)	427 (61)	2.84 (2.22 to 3.64)	<.001	319 (46)	2.57 (1.93 to 3.40)	<.001
	**5-6**	818 (45)	299 (37)	1.69 (1.31 to 2.19)	<.001	249 (30)	1.71 (1.28 to 2.28)	<.001
	**7**	241 (13)	52 (22)	1.00		43 (18)	1.00	
**10 minutes**	**0-2**	145 (8)	100 (69)	3.36 (2.55 to 4.43)	<.001	72 (50)	2.92 (2.10 to 4.05)	<.001
	**3-4**	493 (28)	331 (67)	3.27 (2.52 to 4.25)	<.001	244 (49)	2.91 (2.15 to 3.92)	<.001
	**5-6**	921 (52)	334 (36)	1.77 (1.35 to 2.31)	<.001	271 (29)	1.73 (1.28 to 2.34)	<.001
	**7**	229 (13)	47 (21)	1.00		39 (17)	1.00	

Legend: Shown are absolute numbers (N) of patients with the respective scores and numbers of patients (n). Relative risk (RR), 95%-Confidence Interval (95%-CI) and P-values were calculated for any of the outcome data using the highest score (7) of the *Expanded-Apgar* as reference value.

Additional File 1, Table B. *Expanded-Apgar* and the relative risk of death and perinatal mortality

			Death			Perinatal Mortality		
Expanded-Apgar		**N (%)**	**n (%)**	**RR (95%-CI)**	**P-value**	**n (%)**	**RR (95%-CI)**	**P-value**
**1 minute**	**0-2 points**	54 (3)	13 (24)	7.18 (3.39 to 15.19)	<.001	8 (15)	12.15 (3.79 to 38.95)	<.001
	**3-4**	732 (40)	112 (15)	4.56 (2.49 to 8.36)	<.001	63 (9)	7.06 (2.59 to 19.23)	<.001
	**5-6**	709 (39)	49 (7)	2.06 (1.09 to 3.91)	0.02	17 (2)	1.97 (0.67 to 5.80)	0.21
	**7**	328 (18)	11 (3)	1.00		4 (1)	1.00	
**5 minutes**	**0-2**	74 (4)	23 (31)	8.32 (4.03 to 17.19)	<.001	11 (15)	8.96 (2.94 to 27.29)	<.001
	**3-4**	697 (38)	108 (15)	4.15 (2.14 to 8.06)	<.001	60 (9)	5.19 (1.91 to 14.12)	<.001
	**5-6**	818 (45)	50 (6)	1.64 (0.82 to 3.28)	0.15	18 (2)	1.33 (0.45 to 3.88)	0.60
	**7**	241 (13)	9 (4)	1.00		4 (2)	1.00	
**10 minutes**	**0-2**	145 (8)	28 (19)	5.53 (2.59 to 11.79)	<.001	18 (12)	9.48 (2.84 to 31.60)	<.001
	**3-4**	493 (28)	87 (18)	5.05 (2.49 to 10.24)	<.001	46 (9)	7.12 (2.24 to 22.66)	<.001
	**5-6**	921 (52)	63 (7)	1.96 (0.95 to 4.03)	0.05	25 (3)	2.07 (0.63 to 6.80)	0.21
	**7**	229 (13)	8 (4)	1.00		3 (1)	1.00	

Legend: Shown are absolute numbers (N) of patients with the respective scores and numbers of patients (n). Relative risk (RR), 95%-Confidence Interval (95%-CI) and P-values were calculated for any of the outcome data using the highest score (7) of the *Expanded-Apgar* as reference value.

Additional File 1, Table C. *Expanded-Apgar* and the relative risk for BPD and ROP

			**Bronchopulmonary Dysplasia**			**Retinopathy of Prematurity**		
Expanded-Apgar		**N (%)**	**n (%)**	**RR (95%-CI)**	**P-value**	**n (%)**	**RR (95%-CI)**	**P-value**
**1 minute**	**0-2 points**	54 (3)	14 (26)	2.30 (1.33 to 3.96)	0.003	8 (15)	4.86 (2.01 to 11.76)	<.001
	**3-4**	732 (40)	195 (27)	2.36 (1.70 to 3.27)	<.001	132 (18)	5.91 (3.15 to 11.10)	<.001
	**5-6**	709 (39)	162 (23)	2.03 (1.45 to 2.82)	<.001	80 (11)	3.70 (1.94 to 7.05)	<.001
	**7**	328 (18)	37 (11)	1.00		10 (3)	1.00	
**5 minutes**	**0-2**	74 (4)	29 (39)	5.90 (3.40 to 10.25)	<.001	19 (26)	15.47 (5.43 to 44.04)	<.001
	**3-4**	697 (38)	217 (31)	4.69 (2.88 to 7.63)	<.001	140 (20)	12.10 (4.53 to 32.34)	<.001
	**5-6**	818 (45)	152 (19)	2.80 (1.71 to 4.59)	<.001	70 (9)	5.16 (1.90 to 13.98)	<.001
	**7**	241 (13)	16 (7)	1.00		4 (2)	1.00	
**10 minutes**	**0-2**	145 (8)	56 (39)	4.42 (2.77 to 7.05)	<.001	34 (23)	5.97 (2.95 to 12.07)	<.001
	**3-4**	493 (28)	169 (34)	3.93 (2.54 to 6.07)	<.001	108 (22)	5.57 (2.88 to 10.80)	<.001
	**5-6**	921 (52)	152 (17)	1.89 (1.21 to 2.94)	0.003	74 (8)	2.04 (1.04 to 4.02)	0.03
	**7**	229 (13)	20 (9)	1.00		9 (4)	1.00	

Legend: Shown are absolute numbers (N) of patients with the respective scores and numbers of patients (n). Relative risk (RR), 95%-Confidence Interval (95%-CI) and P-values were calculated for any of the outcome data using the highest score (7) of the *Expanded-Apgar* as reference value.

Additional File 1, Table D. *Expanded-Apgar* and the relative risk for IVH and CPL

			Intraventricular Haemorrhage > Grade 3			Cystic Periventricular Leukomalacia		
Expanded-Apgar		**N (%)**	**n (%)**	**RR (95%-CI)**	**P-value**	**n (%)**	**RR (95%-CI)**	**P-value**
**1 minute**	**0-2 points**	54 (3)	5 (9)	6.07 (1.82 to 20.28)	0.001	5 (9)	3.80 (1.29 to 11.18)	0.01
	**3-4**	732 (40)	37 (5)	3.32 (1.32 to 8.36)	0.006	37 (5)	2.07 (0.98 to 4.40)	0.05
	**5-6**	709 (39)	18 (3)	1.67 (0.62 to 4.45)	0.30	28 (4)	1.62 (0.75 to 3.51)	0.21
	**7**	328 (18)	5 (2)	1.00		8 (2)	1.00	
**5 minutes**	**0-2**	74 (4)	7 (10)	11.40 (2.42 to 53.69)	<.001	1 (1)	0.54 (0.07 to 4.44)	0.56
	**3-4**	697 (38)	40 (6)	6.92 (1.68 to 28.40)	0.001	39 (6)	2.25 (0.96 to 5.24)	0.05
	**5-6**	818 (45)	16 (2)	2.36 (0.55 to 10.18)	0.23	32 (4)	1.57 (0.66 to 3.71)	0.29
	**7**	241 (13)	2 (1)	1.00		6 (3)	1.00	
**10 minutes**	**0-2**	145 (8)	6 (4)	2.37 (0.68 to 8.25)	0.16	2 (1)	0.79 (0.15 to 4.26)	0.78
	**3-4**	493 (28)	34 (7)	3.95 (1.42 to 10.99)	0.003	30 (6)	3.48 (1.24 to 9.77)	0.01
	**5-6**	921 (52)	21 (2)	1.31 (0.45 to 3.77)	0.62	40 (4)	2.49 (0.90 to 6.88)	0.06
	**7**	229 (13)	4 (2)	1.00		4 (2)	1.00	

Legend: Shown are absolute numbers (N) of patients with the respective scores and numbers of patients (n). Relative risk (RR), 95%-Confidence Interval (95%-CI) and P-values were calculated for any of the outcome data using the highest score (7) of the *Expanded-Apgar* as reference value.
