# Supplementary material for: Neonatal assessment in the delivery room – Trial to Evaluate a Specified Type of Apgar (TEST-Apgar)
Source: BMC Pediatr. 2015 Mar 8;15:18. doi: 10.1186/s12887-015-0334-7 (PMC4374498; doi:10.1186/s12887-015-0334-7)
Supplement: Additional file 2: — Specified-Apgar and the relative risk of… (Table E): … poor outcome and morbidity in survivors (page 27), (Table F): … death and perinatal mortality (page 28), (Table G): … BPD and ROP (page 29), (Table H): … IVH and CPL (page 30). [file 12887_2015_334_MOESM2_ESM.pdf]

Additional File 2, Table E *Specified-Apgar* and the relative risk of poor outcome and morbidity in survivors

			**Poor Outcome**			**Morbidity in Survivors**		
Specified-Apgar		**N (%)**	**n (%)**	**RR (95%-CI)**	**P-value**	**n (%)**	**RR (95%-CI)**	**P-value**
**1 minute**	**0-3 points**	321 (17)	212 (66)	1.81 (1.62 to 2.03)	<.001	140 (44)	1.45 (1.24 to 1.70)	<.001
	**4-6**	569 (31)	284 (50)	1.37 (1.22 to 1.54)	<.001	222 (39)	1.30 (1.13 to 1.50)	<.001
	**7-10**	947 (52)	345 (36)	1.00		284 (30)	1.00	
**5 minutes**	**0-3**	56 (3)	43 (77)	1.92 (1.64 to 2.25)	<.001	16 (29)	0.88 (0.58 to 1.33)	0.52
	**4-6**	301 (17)	202 (67)	1.68 (1.52 to 1.86)	<.001	145 (48)	1.48 (1.29 to 1.70)	<.001
	**7-10**	1456 (80)	582 (40)	1.00		475 (33)	1.00	
**10 minutes**	**0-3**	16 (1)	15 (94)	2.16 (1.88 to 2.48)	<.001	5 (31)	0.90 (0.43 to 1.87)	0.77
	**4-6**	104 (6)	71 (68)	1.57 (1.36 to 1.81)	<.001	40 (38)	1.11 (0.86 to 1.43)	0.43
	**7-10**	1662 (93)	722 (43)	1.00		577 (35)	1.00	

Legend: Shown are absolute numbers (N) of patients with the respective scores and numbers of patients (n). Relative risk (RR), 95%-Confidence Interval (95%-CI) and P-values were calculated for any of the outcome data using the highest score (7-10) of the *Specified-Apgar* as reference value.

Additional File 2, Table F: *Specified-Apgar* and the relative risk of death and perinatal mortality

			**Death**			**Perinatal Mortality**		
Specified-Apgar		**N (%)**	**n (%)**	**RR (95%-CI)**	**P-value**	**n (%)**	**RR (95%-CI)**	**P-value**
**1 minute**	**0-3 points**	321 (17)	72 (22)	3.48 (2.54 to 4.78)	<.001	42 (13)	5.63 (3.42 to 9.29)	<.001
	**4-6**	569 (31)	62 (11)	1.69 (1.21 to 2.37)	0.002	31 (5)	2.35 (1.37 to 4.01)	<.001
	**7-10**	947 (52)	61 (6)	1.00		22 (2)	1.00	
**5 minutes**	**0-3**	56 (3)	27 (48)	6.56 (4.73 to 9.10)	<.001	18 (32)	10.88 (6.73 to 17.61)	<.001
	**4-6**	301 (17)	57 (19)	2.58 (1.92 to 3.47)	<.001	32 (11)	3.60 (2.32 to 5.59)	<.001
	**7-10**	1456 (80)	107 (7)	1.00		43 (3)	1.00	
**10 minutes**	**0-3**	16 (1)	10 (63)	7.16 (4.75 to 10.80)	<.001	8 (50)	13.19 (7.64 to 22.78)	<.001
	**4-6**	104 (6)	31 (30)	3.42 (2.45 to 4.77)	<.001	21 (20)	5.33 (3.39 to 8.37)	<.001
	**7-10**	1662 (93)	145 (9)	1.00		63 (4)	1.00	

Legend: Shown are absolute numbers (N) of patients with the respective scores and numbers of patients (n). Relative risk (RR), 95%-Confidence Interval (95%-CI) and P-values were calculated for any of the outcome data using the highest score (7-10) of the *Specified-Apgar* as reference value.

Additional File 2, Table G. *Specified-Apgar* and the relative risk of BPD and ROP

			**Bronchopulmonary Dysplasia**			**Retinopathy of Prematurity**		
Specified-Apgar		**N (%)**	**n (%)**	**RR (95%-CI)**	**P-value**	**n (%)**	**RR (95%-CI)**	**P-value**
**1 minute**	**0-3 points**	321 (17)	98 (31)	1.76 (1.42 to 2.19)	<.001	57 (18)	2.03 (1.48 to 2.77)	<.001
	**4-6**	569 (31)	152 (27)	1.54 (1.27 to 1.87)	<.001	94 (17)	1.88 (1.43 to 2.48)	<.001
	**7-10**	947 (52)	164 (17)	1.00		83 (9)	1.00	
**5 minutes**	**0-3**	56 (3)	11 (20)	0.95 (0.55 to 1.62)	0.84	9 (16)	1.44 (0.78 to 2.66)	0.25
	**4-6**	301 (17)	92 (31)	1.47 (1.21 to 1.80)	<.001	56 (19)	1.66 (1.26 to 2.19)	<.001
	**7-10**	1456 (80)	302 (21)	1.00		163 (11)	1.00	
**10 minutes**	**0-3**	16 (1)	4 (25)	1.14 (0.48 to 2.67)	0.77	3 (19)	1.54 (0.55 to 4.29)	0.42
	**4-6**	104 (6)	25 (24)	1.09 (0.77 to 1.56)	0.62	17 (16)	1.34 (0.85 to 2.11)	0.21
	**7-10**	1662 (93)	365 (22)	1.00		203 (12)	1.00	

Legend: Shown are absolute numbers (N) of patients with the respective scores and numbers of patients (n). Relative risk (RR), 95%-Confidence Interval (95%-CI) and P-values were calculated for any of the outcome data using the highest score (7-10) of the *Specified-Apgar* as reference value.

Additional File 2, Table H. *Specified-Apgar* and the relative risk of IVH and CPL

			Intraventricular Haemorrhage> Grade 3			Cystic Periventricular Leukomalacia		
Specified-Apgar		**N (%)**	**n (%)**	**RR (95%-CI)**	**P-value**	**n (%)**	**RR (95%-CI)**	**P-value**
**1 minute**	**0-3 points**	321 (17)	21 (7)	2.48 (1.41 to 4.37)	0.001	10 (3)	0.74 (0.37 to 1.46)	0.37
	**4-6**	569 (31)	19 (3)	1.26 (0.70 to 2.28)	0.43	27 (5)	1.12 (0.70 to 1.81)	0.63
	**7-10**	947 (52)	25 (3)	1.00		40 (4)	1.00	
**5 minutes**	**0-3**	56 (3)	4 (7)	2.54 (0.94 to 6.84)	0.06	1 (2)	0.42 (0.06 to 2.97)	0.36
	**4-6**	301 (17)	17 (6)	2.01 (1.16 to 3.48)	0.01	14 (5)	1.09 (0.62 to 1.92)	0.76
	**7-10**	1456 (80)	41 (3)	1.00		62 (4)	1.00	
**10 minutes**	**0-3**	16 (1)	2 (13)	4.00 (1.06 to 15.01)	0.03	1 (6)	1.44 (0.21 to 9.75)	0.70
	**4-6**	104 (6)	7 (7)	2.15 (1.00 to 4.62)	0.04	4 (4)	0.89 (0.33 to 2.38)	0.81
	**7-10**	1662 (93)	52 (3)	1.00		72 (4)	1.00	

Legend: Shown are absolute numbers (N) of patients with the respective scores and numbers of patients (n). Relative risk (RR), 95%-Confidence Interval (95%-CI) and P-values were calculated for any of the outcome data using the highest score (7-10) of the Specified-Apgar as reference value.
