# Supplementary material for: Neonatal assessment in the delivery room – Trial to Evaluate a Specified Type of Apgar (TEST-Apgar)
Source: BMC Pediatr. 2015 Mar 8;15:18. doi: 10.1186/s12887-015-0334-7 (PMC4374498; doi:10.1186/s12887-015-0334-7)
Supplement: Additional file 3: — Combined-Apgar and the relative risk of… (Table I): … poor outcome and morbidity in survivors (page 31), (Table J): … death and perinatal mortality (page 32), (Table K): … BPD and ROP (page 33), (Table L): … IVH and CPL (page 34). [file 12887_2015_334_MOESM3_ESM.pdf]

Additional File 3, Table I. ***Combined-Apgar*** **and the relative risk of poor outcome and morbidity in survivors**

			**Poor Outcome**			**Morbidity in Survivors**		
Combined-Apgar		**N (%)**	**n (%)**	**RR (95%-CI)**	**P-value**	**n (%)**	**RR (95%-CI)**	**P-value**
**1 minute**	**0-5 points**	122 (7)	82 (67)	2.27 (1.90 to 2.70)	<.001	52 (43)	1.64 (1.28 to 2.10)	<.001
	**6-9**	439 (24)	272 (62)	2.09 (1.81 to 2.41)	<.001	193 (44)	1.69 (1.43 to 2.01)	<.001
	**10-13**	651 (36)	289 (44)	1.50 (1.29 to 1.74)	<.001	235 (36)	1.39 (1.17 to 1.65)	<.001
	**14-17**	597 (33)	177 (30)	1.00		155 (26)	1.00	
**5 minutes**	**0-5**	31 (2)	25 (81)	2.49 (2.05 to 3.03)	<.001	9 (29)	1.05 (0.60 to 1.84)	0.85
	**6-9**	220 (12)	161 (73)	2.26 (2.00 to 2.55)	<.001	105 (48)	1.73 (1.46 to 2.06)	<.001
	**10-13**	588 (33)	321 (55)	1.69 (1.50 to 1.90)	<.001	255 (43)	1.57 (1.37 to 1.81)	<.001
	**14-17**	951 (53)	308 (32)	1.00		262 (28)	1.00	
**10 minutes**	**0-5**	8 (1)	8 (100)	3.02 (2.78 to 3.29)	<.001	2 (25)	0.91 (0.27 to 3.02)	0.87
	**6-9**	115 (7)	80 (70)	2.10 (1.81 to 2.44)	<.001	43 (37)	1.36 (1.05 to 1.75)	0.02
	**10-13**	512 (29)	334 (65)	1.97 (1.77 to 2.19)	<.001	259 (51)	1.84 (1.61 to 2.09)	<.001
	**14-17**	1103 (63)	365 (33)	1.00		304 (28)	1.00	

Legend: Shown are absolute numbers (N) of patients with the respective scores and numbers of patients (n). Relative risk (RR), 95%-Confidence Interval (95%-CI) and P-values were calculated for any of the outcome data using the highest score (14-17) of the *Combined-Apgar* as reference value.

Additional File 3, Table J. ***Combined-Apgar*** **and the relative risk of death and perinatal mortality**

			**Death**			**Perinatal Mortality**		
Combined-Apgar		**N (%)**	**n (%)**	**RR (95%-CI)**	**P-value**	**n (%)**	**RR (95%-CI)**	**P-value**
**1 minute**	**0-5 points**	122 (7)	30 (25)	6.67 (3.99 to 11.16)	<.001	21 (17)	20.55 (7.90 to 53.45)	<.001
	**6-9**	439 (24)	79 (18)	4.88 (3.09 to 7.71)	<.001	41 (9)	11.15 (4.44 to 27.99)	<.001
	**10-13**	651 (36)	54 (8)	2.25 (1.39 to 3.65)	<.001	25 (4)	4.59 (1.77 to 11.90)	<.001
	**14-17**	597 (33)	22 (4)	1.00		5 (1)	1.00	
**5 minutes**	**0-5**	31 (2)	16 (52)	10.67 (6.86 to 16.61)	<.001	13 (42)	24.93 (13.16 to 47.20)	<.001
	**6-9**	220 (12)	56 (25)	5.26 (3.67 to 7.55)	<.001	29 (13)	7.83 (4.33 to 14.17)	<.001
	**10-13**	588 (33)	66 (11)	2.32 (1.62 to 3.33)	<.001	32 (5)	3.23 (1.79 to 5.84)	<.001
	**14-17**	951 (53)	46 (5)	1.00		16 (2)	1.00	
**10 minutes**	**0-5**	8 (1)	6 (75)	13.56 (8.49 to 21.67)	<.001	5 (63)	31.34 (15.91 to 61.71)	<.001
	**6-9**	115 (7)	37 (32)	5.82 (4.06 to 8.34)	<.001	26 (23)	11.34 (6.64 to 19.34)	<.001
	**10-13**	512 (29)	75 (15)	2.65 (1.92 to 3.65)	<.001	36 (7)	3.53 (2.10 to 5.93)	<.001
	**14-17**	1103 (63)	61 (6)	1.00		22 (2)	1.00	

Legend: Shown are absolute numbers (N) of patients with the respective scores and numbers of patients (n). Relative risk (RR), 95%-Confidence Interval (95%-CI) and P-values were calculated for any of the outcome data using the highest score (14-17) of the *Combined-Apgar* as reference value.

Additional File 3, Table K. ***Combined-Apgar*** **and the relative risk of BPD and ROP**

			**Bronchopulmonary Dysplasia**			**Retinopathy of Prematurity**		
Combined-Apgar		**N (%)**	**n (%)**	**RR (95%-CI)**	**P-value**	**n (%)**	**RR (95%-CI)**	**P-value**
**1 minute**	**0-5 points**	122 (7)	34 (28)	1.91 (1.35 to 2.70)	<.001	21 (17)	2.70 (1.65 to 4.44)	<.001
	**6-9**	439 (24)	129 (29)	2.02 (1.58 to 2.57)	<.001	85 (19)	3.04 (2.12 to 4.37)	<.001
	**10-13**	651 (36)	154 (24)	1.62 (1.28 to 2.06)	<.001	83 (13)	2.00 (1.39 to 2.89)	<.001
	**14-17**	597 (33)	87 (15)	1.00		38 (6)	1.00	
**5 minutes**	**0-5**	31 (2)	6 (19)	1.24 (0.59 to 2.57)	0.57	7 (23)	3.16 (1.58 to 6.30)	0.001
	**6-9**	220 (12)	70 (32)	2.03 (1.59 to 2.59)	<.001	44 (20)	2.80 (1.97 to 3.97)	<.001
	**10-13**	588 (33)	176 (30)	1.91 (1.58 to 2.32)	<.001	106 (18)	2.52 (1.89 to 3.36)	<.001
	**14-17**	951 (53)	149 (16)	1.00		68 (7)	1.00	
**10 minutes**	**0-5**	8 (1)	2 (25)	1.63 (0.49 to 5.46)	0.44	2 (25)	3.40 (1.01 to 11.51)	0.058
	**6-9**	115 (7)	29 (25)	1.65 (1.17 to 2.32)	0.006	21 (18)	2.49 (1.60 to 3.86)	<.001
	**10-13**	512 (29)	183 (36)	2.33 (1.95 to 2.80)	<.001	113 (22)	3.01 (2.30 to 3.92)	<.001
	**14-17**	1103 (63)	169 (15)	1.00		81 (7)	1.00	

Legend: Shown are absolute numbers (N) of patients with the respective scores and numbers of patients (n). Relative risk (RR), 95%-Confidence Interval (95%-CI) and P-values were calculated for any of the outcome data using the highest score (14-17) of the *Combined-Apgar* as reference value.

Additional File 3, Table L. *Combined-Apgar* and the relative risk of IVH and CPL

			Intraventricular Haemorrhage > Grade 3			Cystic Periventricular Leukomalacia		
Combined-Apgar		**N (%)**	**n (%)**	**RR (95%-CI)**	**P-value**	**n (%)**	**RR (95%-CI)**	**P-value**
**1 minute**	**0-5 points**	122 (7)	9 (7)	5.51 (2.17 to 13.98)	<.001	4 (3)	1.09 (0.37 to 3.16)	0.87
	**6-9**	439 (24)	17 (4)	2.89 (1.26 to 6.64)	0.008	22 (5)	1.66 (0.90 to 3.06)	0.09
	**10-13**	651 (36)	30 (5)	3.44 (1.59 to 7.44)	<.001	33 (5)	1.68 (0.96 to 2.95)	0.06
	**14-17**	597 (33)	8 (1)	1.00		18 (3)	1.00	
**5 minutes**	**0-5**	31 (2)	2 (7)	3.61 (0.87 to 14.94)	0.06	0 (0)	0 (0.00)	
	**6-9**	220 (12)	16 (7)	4.07 (2.09 to 7.93)	<.001	9 (4)	1.08 (0.53 to 2.21)	0.83
	**10-13**	588 (33)	26 (4)	2.47 (1.35 to 4.52)	0.002	32 (5)	1.44 (0.90 to 2.29)	0.12
	**14-17**	951 (53)	17 (2)	1.00		36 (4)	1.00	
**10 minutes**	**0-5**	8 (1)	1 (13)	5.74 (0.88 to 37.48)	0.04	0 (0)	0 (0.00)	
	**6-9**	115 (7)	7 (6)	2.80 (1.23 to 6.35)	0.01	3 (3)	0.74 (0.23 to 2.35)	0.60
	**10-13**	512 (29)	28 (6)	2.51 (1.47 to 4.29)	<.001	34 (7)	1.88 (1.20 to 2.94)	0.005
	**14-17**	1103 (63)	24 (2)	1.00		39 (4)	1.00	

Legend: Shown are absolute numbers (N) of patients with the respective scores and numbers of patients (n). Relative risk (RR), 95%-Confidence Interval (95%-CI) and P-values were calculated for any of the outcome data using the highest score (14-17) of the *Combined-Apgar* as reference value.
